# Religious Practices and Its Impacts on a Sustainable Urban Environment in Nigeria: The Way Forward

**DOI:** 10.1155/2023/8080235

**Published:** 2023-11-29

**Authors:** Timothy Oyebamiji Ogunbode, Funke Elizabeth Oyekan

**Affiliations:** ^1^Environmental Management and Crop Production Unit, College of Agriculture, Engineering and Science, Bowen University, Iwo, Nigeria; ^2^Religious Studies and Philosophy Programme, College of Liberal Studies, Bowen University, Iwo, Nigeria

## Abstract

A sustainable and serene environment is a prerequisite and a driver of human productivity in space and time. The quality of a human environment, however, is under threat always partly as a result of various human cultural activities such as festivities and religious practices. This work specifically investigated various ways religious activities have impacted the urban environment in two cities in the southwestern part of Nigeria together as a case study. Data for the work were generated through the administration of 250 copies of the structured questionnaire, out of which 232 were completed and retrieved. The data were subjected to data adequacy tests (i.e., Kaiser–Meyer–Olkin (KMO) and Bartlett's test of sphericity (BTS)). The test showed 67.6% at a significance level of *p* < 0.001 The results showed that 52.6% were of secondary school level, 52.2 were of Christian faith, and 61.6% were also of the male gender who were mostly in the age category of 46 to 65 years. The factor analysis identified 11 out of 23 variables as significant ones which explained 72.052% of the ways religious activities have negatively impacted the urban environment. Of the 11 variables, the first six alone offered 45.248%, namely, (i) contribution to climate change, (ii) night worships, (iii) noise pollution caused, (iv) use of the public address system, (v) quest for expansion, and (vi) religious uses of natural resources, especially water. In view of this, it is recommended that efforts should be made to check the extracted variables for a sustainable serene environment. However, there is a need to put in place relevant legislation/policies to control the activities of various religious bodies and mechanisms to enlighten religious practitioners on practices that will not jeopardize a serene urban environment. Further studies in other places to affirm or otherwise the findings here are suggested.

## 1. Introduction

A sustainable and serene human environment is a strong driver of human productivity in space and time [1–3]. It is not surprising that a sustainable city forms one of the targets listed in the Sustainable Development Goals (SDGs) by 2030. However, various anthropogenic and cultural activities exacerbate negative influences and impacts on the human environment, which if not curtailed, may hinder the realization of the set target [4, 5]. Some anthropogenic activities in this category include social events, traditional festivals, religious activities, and small and medium industries. The role of religion cannot be overruled in bringing people together and giving a sense of belonging, thereby impacting depression minimization and growth of society, most specifically in the Sub-Saharan region [4, 6, 7]. In the African context and Nigeria, in particular, religion is very salient in the development of various communities regardless of whether traditional, Christianity, or Islam. Religions have contributed immensely to the growth of African society in the areas of health promotion, growth in education, infrastructural development, harmonious existence of people, and so on [8, 9].

The practice of religious rites has almost become a strong determinant of every action of man in Africa that a man's plan can hardly be divulged from his religious beliefs [6, 7]. Africans, generally, could be ready to lay down their life for the sake of defending their religion [9]. This attitude has, however, partly impacted negatively on many communities and individuals such that its relevance in being a succor and attraction is diminishing [7]. For instance, the current insecurity situation in Nigeria was partly premised on the need to enforce a particular religion on the citizens [10, 11]. Apart from this, the rate of expansion of different religions in Nigeria, especially Christianity and Islam, is becoming alarming because their actions portend discomfort and pollution to the built environment [12–14]. Furthermore, the rate at which natural resources are depleted in the name of religion calls for concern [15, 16]. Natural resources have been at the receiving end as a result of vegetal removal, destruction of the ecosystem, indiscriminate location of their respective worship centres, use of bells, actions that induce noise pollution, cemetery land use, urban planning distortion, and many menaces [17, 18]. Worship venues and centres are found almost in every corner of cities and villages. According to Adebayo [17], this action has become so terrible that within a square metre, there could be multiple Christian and Muslim worship centres which are of diverse denominations. For example, Baptists, Redeemers, Anglicans, and Methodists of Christian faith and Nurudeen, Anwar Ul, and Ahmadiyyah all of Muslim faith could be within such a small space to the extent that residential buildings have been tending towards being swallowed by all these religious activities [17]. All these religious impacts are felt by all other components of the economy in several ways. It is from this perspective that this investigation has been carried out to assess various ways all these religious bigotries and sentiments have impacted on the livelihood in urban centres. The objectives of the study are (i) identifying various ways religious activities have impacted the urban living environment, (ii) quantifying the various identified variables in (i), and (iii) ranking the identified variables in order of their respective weights as they impacted on the urban livelihood.

## 2. Method of Study

### 2.1. Study Area

**Figure 1:**
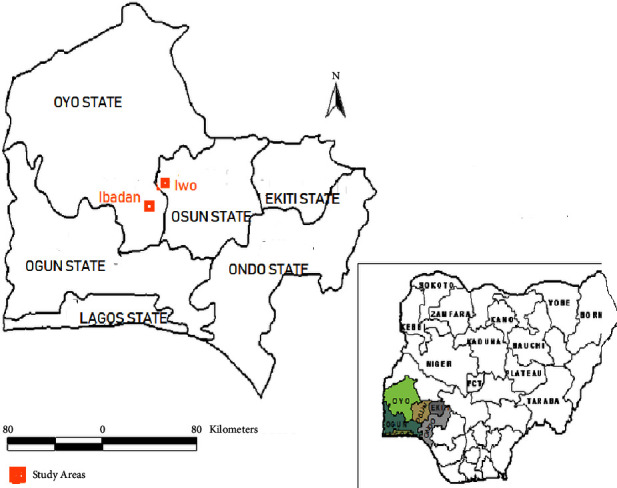
Map of southwestern Nigeria showing Iwo and Ibadan (inset: map of Nigeria showing the southwestern region (source: Google Maps).

The study was carried out in the southwestern part of Nigeria comprising two adjacent towns in two neighbouring states, namely, Iwo (Osun state) and Ibadan (Oyo state) (Figure 1). Iwo town was projected to have a population of 263,500, while Ibadan was projected to have a population of 1,887,100 by 2022 [19] and has a population of 1,887,100. Southwestern Nigeria comprises six states, namely, Lagos, Ogun, Ondo, Oyo, Osun, and Ekiti, all forming a geopolitical zone of the six zones in the Federal Republic of Nigeria. The Yoruba tribe forms the dominant tribe. Major occupations in the zone include farming, business, and education. The region is dominantly tropical with two distinct seasons, namely, dry and wet seasons. The wet season spans from March through October, while the dry season commences from November through February. Dominant vegetation is tropical comprising hardwoods such as Iroko, Mahogany, and Obeche. However, most parts of this natural forest have been cleared for farming purposes; thus, secondary forest now predominates in the region. Towards the northern part of the region is derived savannah and the extreme south is found swampy vegetation. Most inhabitants of this region are of Christian, Muslim, or traditional faiths. Thus, the region comprises churches, mosques, and shrines for their respective worshipers. The southwestern part of Nigeria is one of the regions in the country that experiences tremendous expansion of religious activities to the extent that there is hardly a settlement, regardless of its size, which does not have the footprint/s of religious activities [20–22]. In fact, a detailed explanation of infrastructural development in various cities and villages will be insufficient without recourse to religious influence [20]. Religions have become so dominant in human endeavours and philosophy that they dictate almost every action and thought of the inhabitants [23, 24]. According to Johnson and Zurlo [25], Islamic religious groups formed 46.2%, Christianity formed 46.3%, and the other religions were estimated to be 7.5% in Nigeria. The number of Christian and Muslim worship centres is still scary in the literature, but research has shown that the number of church buildings in Nigeria is increasing at an alarming rate [26]. According to Ogunbade, worship centres have been multiplying in Nigeria, the situation he attributed to the constitutional entrenchment of freedom of religion, high rate of unemployment, love of money and wealth, love of a position of authority, economic downturn, and unhealthy rivalry between Christianity and Islam. Thus, it was noted that the establishment of churches is being portrayed as a business venture. More importantly, the fact that worship centres are free from tax payment also contributed to the scenario.

### 2.2. Data Collection

Research data were generated through the administration of questionnaires in the study area. Two hundred and thirty-two (232) copies of the questionnaire were administered across randomly selected households from the five quarters into which the town is divided. The survey was biased against adults who are household heads. Forty-four structured questions which were subdivided into Sections A and B. Section A consisted of questions about the details of the respondent such as name, age, and religion. Section B contained short structured questions concerning various ways in which religion has impacted on the environment from every respondent's perspective. Observations were equally made during the administration to complement the information generated through the survey. It should be noted that no formal ethical code was required for the administration of the questionnaire.

### 2.3. Data Analysis

Both descriptive and inferential statistical analyses were carried out. Descriptive analysis involved the use of tables and percentages, while inferential analysis involved the use of exploratory factor analysis to extract various factors that explained how religion has influenced the quality of an urban environment. The use of factor analysis is not new in environmental investigations; for instance, Hopke [27] and Ogunbode et al. [28, 29] had successfully used the method in environmental studies and have found it robust for use.

## 3. Results and Discussion

### 3.1. Descriptive Analysis

Table 1 shows some of the attributes of the respondents.

Table 1 shows that respondents within the category of 46 to 65 years formed the majority of 54.7%, while most of the respondents (52.6%) had secondary level of education. This is not surprising because the survey considered the ability of the potential respondents to read and understand the questionnaire and also be able to respond appropriately. In addition, 52.2% and 43.5% of the respondents were of Christian and Islamic faiths, respectively, while the traditional religionists accounted for 4.3%, indicating the peculiarity of the study area in terms of religious beliefs. Traditional religion is less popular when compared with the other two faiths in Yorubaland where the study was conducted. Another feature of the respondents was the dominance of male gender in the survey. Investigations have shown that men's faith often influences women's faith at the point of marriage. Thus, a man's faith becomes a woman's faith as long as she remains under the roof of the man as his wife. Nwufor and Otor [30], Udoh et al. [31], and Kyei [32] had all observed among Africans that the culture that subjugates women under men in terms of religion and marriage has impacted on their desire of leadership. It was on the premise of this fact that the survey focused on male gender and only where the man was not available, the woman in the household was involved in the survey.

### 3.2. Impacts of Religious Activities on Urban Environment Quality

The data generated were subjected to data adequacy (i.e., Kaiser–Meyer–Olkin (KMO) and the Bartlett's test of sphericity (BTS) showed 67.6% at a significance level of *p* < 0.001 indicating that the data are factorable and free of collinearity among the dataset. An eigenvalue was also set at a minimum of 1.000. This means that any variable with less than the set minimum is not considered as strong enough to contribute as an explanatory variable. In addition, for inclusion in the determining variable in the rotated component matrix (RCM), the variable should have the highest percentage in the array of the factors as generated by FA.

The results of factor analysis (FA) are presented in Table 2.

Eleven variables (11) were identified and extracted as significant ways by which religious activities have impacted on the environment under study.

The first and only variable with the highest weight of the 11 variables was the religious contribution to climate change. It is ranked first with an eigenvalue of 2.630, and its weight from the total explanation was 8.765% out of 72.052%. The impacts of religious activities on climate change range from the removal of vegetation to their effect on changing the natural soil with concrete and exposure of soil to direct sunshine [33] and also influence the perception and the level of their involvement of their members in mitigating or otherwise of the menace. Haluza-DeLay [34] had recommended that climate change social scientists should close ranks with religious institutions in order to check the menace; moreover, such religious institutions are often major social institutions and action collection media which would be beneficial to climate change scientists.

Following this variable is the religious night worship, otherwise called night vigil. It is ranked second with an eigenvalue of 2.53, and its proportion from the total weight was 8.447%. The culture of holding night vigils, most especially, by the Christian faith has been identified as a way of fulfilling the tenets of one of the injunctions in the Bible [35]. The impact of this practice has been identified by [17] and described it as being detrimental to human health and comfort, especially for those that might be nursing one or other health challenges. The challenge emanating from this practice has been further compounded by the indiscriminate location of different worship centres within the residential areas of the town [36]. Gbadegesin and Adeyemi-Adejolu [37] identified some of the challenges arising from vigils to include economic hours are spent looking for miracles through night vigils and closure of functional microbusiness. Thus, the poverty level is becoming aggravated.

Also identified as one of the ways religious activities have impacted the quality of the built environment was noise pollution caused by these religious groups in the study area. It is ranked third with an eigenvalue of 2.395 and a weight of 7.840%. Religious activities are perhaps associated with the creation of noise and may contribute to human discomfort in the neighborhood. Very common in worship places is the use of the public address system (PAS) which is used to amplify noise. Noise pollution has been identified by Oyati and Stephen [38] and Abolade et al. [36] as one of the areas through which religious activities exert degradation to the environment. Some researchers believe that noise associated with worship centres should be mitigated because of their negative impacts on man and the environment [36, 38, 39], while others revealed that the noise has no negative impact on the environment, e.g., [40]. These views, notwithstanding, report that noise is a negative feature and should be checked in the human environment. For instance, Oloruntoba et al. [41] and Wokekoro [42] identified the health challenge posed by noise pollution for humans. According to him, cases of headache, stroke, and a rise in blood pressure are all attributed to the degraded quality of life caused by noise pollution. Adesoji [22] had reported that these illnesses are on the rise in Nigeria.

The fourth variable with an eigenvalue of 2.311, which was identified with a significant weighted value of 7.704%, was the environmental effect of the loudspeakers or the public address system (PAS) being used by different religious groups in the study area. There is hardly any religious group that does not amplify their religion with the aid of PAS. Dickson [43] expressed his concern over the noise generated from worship places as a result of the use of loudspeakers which includes sleep disturbance and a potential source of aggravation of blood pressure and so suggested that sanctions should be instituted against any erring religious bodies. However, Akintaro [40] found no significant apparent effect on human health.

The next factor identified as another way religious activities have caused degradation to the environment was the quest and the pattern of expansion of different religious groups in the study area. This variable was ranked fifth with an eigenvalue of 1.923 of the 11 variables extracted. Its proportion from the total weight was 6.410%. Although there is a dearth of figures to show the trend in the expansion, observations showed that the quests of different religious groups for expansion in order to add to their followers and also to keep the existing members within their respective folds have led to indiscriminate location of worship centres within nooks and cranes of residential places in the town. It was further noticed that both Christian and Muslim religious worship centres are located within a few metres apart, even when the members are so few [44]. Odunola et al. [45] observed a similar trend in Ibadan where he noted unguided noncompliance to the rule in the location of worship centres in Ibadan. Also adding to this is the removal of vegetation for new worship centres, especially at the outskirts of the town, for potential and existing members who might have moved to their new houses located at that location [46]. Ogunbode et al. [29] corroborated this fact from their findings in Ibadan. Thus, the pattern of expansion being exhibited by these religious groups has the tendency to aggravate the rate of degradation of the human environment if not checked.

The sixth variable extracted by FA was the religious use/s of water. This was ranked sixth of the 11 factors with an eigenvalue of 1.780. It contributed 5.935% of the total weight of 72.052%. The use of water forms part of different religious groups. For instance, the use of water for ablution purposes characterizes the worship needs of Islam. Often, water is provided in each of the worship centres for ease of access for worshipers. In the Christian faith, some denominations believe in the use of water for spiritual cleansing/baths and other “miracles.” Most often, their use of water leads to unwarranted odour and poor quality of the environment as a result of effluents from all these worship places. Apart from this, surface waters are superfluously polluted through baths in streams. Mujawar et al. [47] made similar observations in India when he discovered that water quality was distorted as a result of religious activities at the temple area. Okolo [46] lamented the rate at which the beauty of the built environment is being degraded as a result of the undiscerning use of natural resources (water inclusive), by religions in Nigeria, and so, recommended the formulation of appropriate policies to forestall the habit. The use of water in various worship practices also makes the resource valuable and expendient to both Christians and Muslims. Muslims use water for ablution purposes [48], while Christian denominations such as Baptists, Celestials, Cherubim, and Seraphim use water as objects for miracles and for baptismal services. The quality of the environment may be at the receiving end of using this resource for various purposes, which may have to be checked [49].

The seventh way by which religious practices had inflicted on the quality of the built environment in Iwo is what these religious groups portend about environmental preservation. With an eigenvalue of 1.767, this variable presented 5.090% of the total weight offered by the 11 extracted variables. The response of the respondents showed that while some believe that the natural environment should be protected by humans, some believed that it is God who orders how the environment is at any point in time. Asu [50] and Onnoghen et al. [51] found that there was a correlation between religions, especially Christianity and Islam, and resource conservation which was otherwise stated to imply that God, the creator of the universe, is against recklessness and wastage of natural resources or any act that could jeopardize the continuous existence of these natural endowments.

The length of stay of a particular religious group was also regarded as a significant way by which all these religious sects impacted on the physical environment. It is ranked eighth with an eigenscore of 1.749 and a weight value of 5.828%. The longer the stay of any of these religious groups in a particular location, the more intense are the challenges caused by their various activities when such activities remain unchecked, especially through the bad odour initiated by effluents discharged into the environment, noise pollution, and water contamination in the environment. Adebayo [17] in support of this finding revealed that religious activities had reported that the continuous existence of religious groups in a given locality had led to the persistent and uncontrolled negative impact on environmental quality. However, Akintaro [40] and Chuvieco et al. [52] made observations which were, rather, contrary to this finding, having found that religion demonstrated little explanatory power to the environmental performance. In fact, Akintaro [40] stressed that the noise associated with religious activities had no effect on human health.

FA identified current challenges created by climate change scenario as one of the ways a religious group in the study area has impacted on the quality of the urban environment. This variable was ranked ninth on the array of the extracted variables with an eigenvalue of 1.557 and 5.255% of the total variance. The challenge of climate change which has resulted in unpredictable rainfall patterns, increasing temperature, poor cropping, and also environmental pollution cannot be fully explained without adequate reference to the activities of religions in the study area. Hope and Jones [53], Jenkins et al. [33], and Ogunbode [26] had noted that the contributions of religious views and perceptions about climate change need to be understood in order to understand how they contribute to the scenario. It is significant that all hands must be on deck for a sustainable religious practice without inflicting negative impact on the sustainable environmental quality in order to attain sustainable living. Posas [54] and Sayem [55] had also observed that involvement and influence on climate change issues on the basis of religion portend significant future relevance of religion in solving crisis associated with climate change.

The threatening status of religious activities to the quality of the human environment was also extracted and ranked tenth in the list of the extracted factors. It has an eigenscore value of 1.527 and presented a weight of 5.091% of the total explanation. The indiscriminate location of worship centres, the release of effluents through the use of water, surface water contamination, noise pollution, and diversion of various public places for religious purposes could jeopardize sustainable human living in the study area if not checked through the promulgation of relevant policies by stakeholders. Though bigotry and religiosity may blindfold those that practice these religions, long-term physical implications may not be quantifiable in terms of health, socioeconomic, and environmental implications. Adebayo [17] and Hope and Jones [53], in corroborating this discovery, bemoaned the abuse inflicted on the environment through the activities of various religious practitioners which have led to the degraded quality of the environment. Negi [56], however, recommended that religion should realign to suit towards its potential roles in ensuring sustainable resource conservation.

The last of the eleventh variable that offered an explanation on the subject matter was the claim of the respondents on the religious impacts on natural resources such as water, vegetation, soil, and the atmosphere. The extraction of this factor has actually substantiated the general claim that religious activities added no value to general environmental resources. Notwithstanding, it was discovered that rather than improving the quality of these natural endowments, most religions carry out their activities mostly on the belief that the resources and every other thing belong to a supreme being who is in charge of the quality and caretaking of the universe in its entirety. Hope and Jones [53] and Schuman et al. [57], in supporting this view, lamented the danger inherent in the lackadaisical attitude of most religions towards human environmental stewardship on the note that nature should take care of itself when a man persistently and uncontrollably encroaches and utilizes nature. There is still a dearth of information on the proportion of the contributions of religious bodies to the degradation of natural resources in Nigeria. However, the Land Degradation Neutrality Programme [58] discussed resource degradation in Nigeria and identified eight hotspots that call for remediation which covers ten states. For instance, the degraded area in Oyo state covers about 66.29 hectares, while that of Zamfara state is 65.25 hectares. These two states are, respectively, largely made up of Christianity and Islamic beliefs.

The quantification of the contributions of religious practices to the quality of the urban environment in this work is a remarkable input to the frontiers of knowledge in Africa and most importantly in Nigeria. This is expected to enhance the understanding of desirable steps towards resolving the menace associated with the challenge of religious practices so as to achieve a sustainable urban environment as being pursued globally through SDG 11.

## 4. Conclusion and Policy Recommendation

Despite the benefits derivable from the activities of various religious activities in Africa and especially in Nigeria, the investigation conducted showed that they still pose some challenges to the human environment. The survey conducted showed that most of the respondents were of male gender and of secondary level of education. From various ways identified by which religious activities had impacted on the study area, eleven variables were found to be significant ways religious activities have inflicted discomfort to the environment. The first factor which had the highest explanation for such impacts was the religious contribution to climate change, while the variable that offered the least explanation was the impact on the quality of natural resources. In view of the findings of this investigation, stakeholders may have to consider further education and enlightenment of various leaders in different religions. Respondents seem to be so glued to their respective religions that their leaders may have to work on and sensitize their respective followers on the significance of religious practices that do not jeopardize sustainable virile and quality environment for improved urban comfort. Initiating appropriate legislation will equally go a long way to check the negative impacts of religious activities in urban centres. Further investigation in other cities is suggested for further understanding of the impacts of religious activities on sustainable urban comfort.

## Figures and Tables

**Table 1:** Pooled demographic attributes of the respondents.

Categorization	Distribution	
	Sample size	% of total in the category
(A) Age category		
18–45 years	92	39.7
46–60 years	127	54.7
>60 years	13	5.6
(B) Level of education		
Primary	56	24.1
Secondary	122	52.6
Tertiary	43	18.5
Others	02	0.9
No formal education	09	3.9
(C) Religious group		
Christianity	121	52.2
Islam	101	43.5
Traditional religion	10	4.3
(D) Gender		
Male	143	61.6
Female	89	38.4

Source: authors' fieldwork.

**Table 2:** Extracted variables and their respective details.

Rank	Variable extracted	Eigenvalue	% explanation	% cumulative explanation
1	Religious contribution to climate change	2.630	8.768	8.768
2	Religious night worship	2.534	8.447	17.215
3	Noise pollution caused by these religious groups	2.395	7.840	25.199
4	Environmental effect of loudspeakers or public address system (PAS)	2.311	7.704	32.903
5	Quest for and the pattern of expansion of different religious groups	1.923	6.410	39.313
6	Religious use/s of water	1.780	5.935	45.248
7	Religious views about environmental preservation	1.767	5.890	51.138
8	Length of stay of a particular religious group	1.749	5.828	56.967
9	Current challenges caused by climate change scenario	1.577	5.255	62.222
10	Respondents' view on the threatening status of religious activities	1.527	5.091	67.313
11	Religious impacts on the natural resources	1.422	4.740	72.052

Source: SPSS factor analysis total explained table.

## Data Availability

The underlying data are embedded in the article.
